# A Review of the Impact of Bariatric Surgery in Women With Polycystic Ovary Syndrome

**DOI:** 10.7759/cureus.10811

**Published:** 2020-10-05

**Authors:** Robert Lee, Christopher Joy Mathew, Merin Tresa Jose, Abeer O Elshaikh, Lisa Shah, Ivan Cancarevic

**Affiliations:** 1 Surgery, California Institute of Behavioral Neurosciences & Psychology, Fairfield, USA; 2 Medicine, California Institute of Behavioral Neurosciences & Psychology, Fairfield, USA; 3 Family Medicine, California Institute of Behavioral Neurosciences & Psychology, Fairfield, USA; 4 Internal Medicine, California Institute of Behavioral Neurosciences & Psychology, Fairfield, USA; 5 Family and Community Medicine, Smt. Nathiba Hargovandas Lakhmichand Municipal Medical College, Ahmedabad, IND

**Keywords:** polycystic ovary syndrome (pcos), hyperandrogenism, bariatric surgery, insulin resistance, obesity

## Abstract

Polycystic ovarian syndrome (PCOS) is the most common endocrine disorder in reproductive-age women that causes infertility. Obesity and insulin resistance are closely tied to the pathophysiology of PCOS. Current first-line treatments include lifestyle modifications, hormone modulators, and laparoscopic ovarian drilling, but little attention has been given to bariatric surgery as a viable option. A detailed review of the literature regarding the outcomes of obese women with PCOS after bariatric surgery is necessary. All studies were found in the PubMed database, limited to females and humans, and selected due to relevancy and quantitative data. Bariatric surgery promotes significant weight loss within one year, which is associated with amelioration of insulin resistance, hyperandrogenism, menstrual irregularity, and ovulatory dysfunction. Surgery successfully mediates the regression of PCOS and promotes successful pregnancy. Thus, we recommend the consideration of bariatric surgery as part of the main treatment considerations in obese patients with PCOS. However, more focused and comprehensive research with better study designs are still needed in the future to investigate PCOS and bariatric surgery.

## Introduction and background

Polycystic ovarian syndrome (PCOS) is one of the most common endocrine disorders that affects 7% to 18% of reproductive-age women in the United States [1,2]. It is also a leading cause of infertility in this population. Stein and Leventhal first classically described PCOS in 1935 [3]. The underlying mechanism of PCOS is complicated with an unknown etiology, but much research has been performed to increase our understanding since then. The World Health Organization (WHO) group 2 is a category of most common causes of normogonadotrophic anovulation, in which PCOS is classified as by far the most prevalent cause [4]. Due to the multi-faceted presentation of PCOS, its diagnosis remains one of exclusion [5]. Per recommendation by the Endocrine Society, the diagnosis of PCOS should be based on the 2003 Rotterdam criteria [6]. According to these criteria, two out of three findings are required: hyperandrogenism (HA), ovulatory dysfunction, and polycystic ovaries confirmed on ultrasound (USG) [7]. Insulin resistance has been consistently tied to patients with PCOS [8]. Ultimately, PCOS manifests clinically as a chronic hyperandrogenic state that leads to multiple short- and long-term conditions, such as oligomenorrhea, hypertension (HTN), infertility, hirsutism, type 2 diabetes mellitus (T2DM), dyslipidemia, and increased risk of endometrial cancer [5, 8]. Various treatments for women with PCOS are available now, but the overall impact on fertility is still under review.

Treatment for PCOS is complicated by its complex pathophysiology. A systematic review and meta-analysis concluded that obesity was more prevalent in women with PCOS as opposed to women without PCOS [9]. Thus, first-line therapy is aimed at lifestyle modifications and weight loss [10]. A reduction in body weight lends itself to improve response to ovulatory induction and hormonal treatments. Due to its relation to insulin resistance, PCOS has been treated with metformin. While postulated that the use of metformin would increase reproductive outcomes in patients, a 2017 Cochrane review suggests that its use versus placebo may improve higher rates of pregnancy and ovulation [11]. However, when compared to clomiphene citrate (CC), a selective estrogen receptor modulator, findings for live births were inconclusive and inconsistent [11]. CC is recognized as the first-line therapy for ovulatory induction in the last 40 years [12]. Studies on the CC stair-step (CC-SS) protocol show a considerable reduction of time to ovulation and favorable reproductive outcome [13]. Surgical therapies, such as laparoscopic ovarian drilling (LOD), have developed that lead to beneficial results for patients, but are not considered viable as a first-line treatment when compared to CC [14,15]. A Cochrane review concluded there was no clear evidence that LOD ameliorates PCOS compared to other medical treatments [16].

Bariatric surgery began in the 1950s with intestinal bypass, but laboratory research began in 1965 when Dr. Edward Mason, the “father of obesity surgery,” developed the original technique [17]. This category of surgery includes different procedures: Roux en Y gastric bypass (RYGB), Sleeve Gastrectomy (SG), and Adjustable gastric band (AGB), among others. These procedures are a form of hormonal surgery, altering gut hormones due to either reduction and/or malabsorption. Long-term studies show that surgery produces significant long-term weight loss, remission of diabetes, and improvement in cardiovascular risk factors (HTN, dyslipidemia) [18]. For an average of six years, a majority of patients having surgery were able to maintain a healthier state [18]. Bariatric surgery is generally well tolerated, with the risk of major complications (anastomotic leaks, bowel obstruction, and others) occurring in an average of ~8% of patients at one year follow up [19]. Increased safety and refinement of these procedures have led to more patients becoming viable candidates for this type of treatment.

Bariatric surgery has been utilized to treat PCOS, but recommendations for its use after failure of other frontline therapies have not been fully established. The position laid out by the American Society for Metabolic and Bariatric Surgery in 2017 stated that while bariatric surgery has been shown in some case-control studies to improve fertility, its specific impact on the responsiveness to subsequent infertility treatments is still not clearly understood at this time [20]. In this article, we will review the current literature to identify studies that evaluate the clinical application of bariatric surgery in the treatment of PCOS.

## Review

The literature used in this review was selected from the PubMed database based on relevancy. English-language articles were identified using the search terms “polycystic ovary syndrome or PCOS” and “bariatric surgery” limited to females and humans. Additional literature was collected through cross-referencing articles in the reference list of selected articles.

Eighty-eight titles were identified through the primary search. Of those titles, a total of 10 studies (six full manuscripts and four abstracts) were selected for review. Overall, 225 women were evaluated for multiple outcomes related to PCOS and bariatric surgery.

Lifestyle and dietary modifications that facilitate weight loss are fundamental first-line management of PCOS. Considering that the benefits of these measures are not typically maintained long-term, bariatric surgery should naturally be incorporated in PCOS treatment options. A systematic review by Buchwald et al. reported excess weight loss (EWL) of 61.2% average for patients of various gastric bypass procedures [21]. These findings are further confirmed by a more recent comparative study written by Carlin AM et al. that similarly reports 69% EWL after RYGB at one-year follow-up [22].

Escobar-Morrale et al. studied 17 women with PCOS that underwent either Scopinaro’s biliopancreatic diversion or laparoscopic gastric bypass [23]. Mean weight loss for the 12 patients available for follow-up was 41±9 kg (95% confidence interval, P < 0.001) after 12±5 months [23]. The weight loss corresponded with a major normalization of total and free testosterone, androstenedione, dehydrogeipiandrosterone sulfate (DHEAS) levels, as well as witnessing an increase in circulating sex hormone-binding globulin (SHBG) [23]. Additionally, the hirsutism (Ferriman-Gallwey) score decreased (from 9.5±6.8 to 4.9±4.2; P=0.001), and insulin sensitivity was also reestablished (from 5.8±2.8 to 1.6±1.0, P<0.001) [23]. The menstrual cycles of patients returned to normal for all patients. In 10 of these patients, ovulation was confirmed restored using measurements of luteal phase serum progesterone concentrations [23].

Eid et al. reported that among 24 patients with PCOS treated by RYGB, there was a mean excess weight loss of 56.7%±21.2% and a mean BMI of 30±4.5 at one-year follow-up [24]. Associated with the weight loss was T2DM resolution in all patients and normalization of HTN (78%) and dyslipidemia (92%) [24]. Of the 23 women who originally exhibited hirsutism characteristics, 12 completely resolved at 8±2.3 months follow up, nine displayed various degrees of resolution postoperatively, while two reported no change [24]. All women reported resumption of normal menstrual cycles after a mean of 3±2.1 months after surgery [24]. Fertility was restored to five women who were able to conceive without the use of CC therapy [24].

Jamal et al. examined outcomes in 20 women after RYGB for a mean of 46.7 months [25]. Preoperative mean BMI was 52.8 ± 9.08 kg/m^2^, and after surgery mean %EWL was 64% by the end of the study [25]. Hirsutism resolved in four (29%) patients, in which improvement was tied to 45% EWL [25]. Metabolic disorders improved with weight loss; seven (77.8%) of the women originally with T2DM experienced complete remission and three (50%) of HTN resolved, most within the first month [25]. Menstrual cycle also normalized in 14 (82%) patients, helping six of ten patients who previously were unable to become pregnant conceive within three years of treatment [25].

Legro et al. published a study of 29 obese women treated with RYGB [26]. Significant weight loss was noted, particularly the ratio of android to gynoid fat declined, at 12 and 24 months follow up [26]. No significant change in hirsutism profile was discovered, but androgen hormone levels did alter substantially at 12 and 24 months [26]. SHBG notably increased within the first month, which correlated to a peak drop in testosterone and estradiol levels at the three- to six-month time frame [26]. Follicular phase length was shortened 7.9-8.9 days postoperatively six months (P<0.001) [26]. Alternatively, menstrual cycle endocrine profiles were found to be quite similar at each follow-up visit with no major change compared to pre-operation levels [26]. Total ovarian volume did not significantly change over 12 months (16.1±13.1 cm^3^ baseline vs. 13.3±6.4 cm^3^ at 12 months, P=0.70) nor in size of largest follicle size (12±9.4 mm baseline vs. 8.6±2.9 at 12 months, P=0.16) [26].

A study by Eid et al. reported a decrease in mean BMI for 14 women from baseline 44.8±5.9 kg/m^2^ to 29.2±5.9 kg/m^2^ 12 months postoperatively [27]. Metabolic markers were seen to benefit from RYGB, with a substantial decrease in fasting glucose (FBS), insulin, cholesterol, and triglyceride levels at 12 months (P<0.05) [27]. Noteworthy improvement was observed in testosterone levels at 12 months [27]. Hirsutism resolved in seven of 11 patients at 12 months, and regular menses was restored within six to 12 months postoperatively to all 10 patients who had irregular cycles at baseline [27]. Interestingly, the study also concluded that the degree of weight change did not correlate to improvements in the results mentioned above.

Wang et al. studied two groups of 24 obese patients with PCOS and compared treatment with laparoscopic SG to that of lifestyle modifications [28]. Body mass and BMI were found to be significantly reduced in the SG patient group three months after surgery, with maximum loss observed at six months after surgery. Comparatively, patients in the SG group showed greater weight loss (P<0.0001) [28]. Androgen levels, on average, dropped significantly following surgery (P=0.012) [28]. More pronounced improvement in restoration of menstrual cycles and ovulation was noted, especially three to six months postoperatively [28].

A more focused study by Turkmen et al. evaluated the metabolic change in 13 obese women with PCOS for six months after undergoing RYGB [29]. At six months after surgery, mean BMI significantly decreased (47.15±7.57 kg/m^2^ baseline vs. 35.46±7.04 kg/m^2^ at six months) and all metabolic syndrome-related biomarkers, minus serum HDL levels and diastolic blood pressure, were normalized [29]. By the end of the study, there were still six anovulatory patients [29]. Testosterone and SHBG normalized in all patients, but serum progesterone and allopregnanolone levels only increased in ovulatory patients [29]. However, total ovarian volume at six months posed no difference between the two patient groups [29]. These findings suggest a relationship between ovulation, progesterone, and its metabolite allopregnanolone [29].

Abiad et al. analyzed the effects of weight loss by SG on CRP and adiponectin among 22 obese women at three, six, and twelve months [30]. BMI among the six obese PCOS patients substantially dropped one year later (36.28%) compared to obese non-PCOS patients (33.04%) (P=0.002) [30]. Both SHBG (58.62±30.44, P=0.005) and total testosterone (10.29±6.30, P=0.011) significantly improved within the first three months, but both also held constant in further months [30]. Insulin levels displayed a downward trend, with marked improvement within the first three months (14.45±7.49, p=0.005) postoperatively, which coincided with a significant decrease in FBS (94.5±9.73 to 85±7.81, P=0.003) [30]. Lipid profile significantly improved, which matched increased adiponectin levels post-surgery (5.05±1.98-7.25±0.21) at all follow-up intervals [30]. Ultimately, decreased CRP levels in association with weight loss was significantly observed for the PCOS group at three months (4.18±3.94, P=0.048), but leveled off in subsequent follow up [30].

Christ and Falcone performed a review on 44 women with PCOS regarding the impact of bariatric surgery on metabolic and hormone levels and its predicted benefits post-operation [31]. Substantial reductions in BMI and lipid profile were noted postoperatively (P<0.05) [31]. Patients’ androgen levels also significantly decreased (P<0.05) such that the percentage meeting the criteria for hyperandrogenism and irregular menses subsequently dropped (P<0.05) [31]. However, ovarian volume (OV) was not seen as significantly declining postoperatively [31]. In the study, analyses showed that preoperative OV was the best predictor of change in HbA1c (β 95% (confidence interval) 0.202 (0.011-0.393), P = 0.04) and triglyceride (6.681 (1.028-12.334), P = 0.03), while free testosterone was indicative of change in total cholesterol (3.744 (0.906-6.583), P = 0.02) and non-HDL-C (3.125 (0.453-5.796), P = 0.03) levels [31].

In the latest study by Singh et al., 18 women diagnosed with PCOS displayed increasing amounts of weight loss within a one year follow-up time frame [32]. %EWL at three months, six months, and one-year follow-up was 31%, 49%, and 63%, respectively, among patients [32]. Patients with metabolic syndrome benefited from weight loss as all cases resolved by the end of the study period [32]. Mean serum testosterone decreased (baseline 0.083±0.38 ng/ml to 0.421±0.25 ng/ml, P<0.01) at one-year follow-up; however, changes in serum LH and FSH were insignificant. Hirsutism completely resolved in 44% (5/11) with a significant decrease in mean score from 11 to 9 after one year's time [32]. Preoperatively, 77% (14/18) of women presented with polycystic ovaries on USG, and post-surgery 55% showed complete resolution over one-year follow-up period [32]. This finding corresponded with all females regaining normal menstrual cycle function by three months' time of follow-up [32].

Weight loss and bariatric surgery

Obesity and PCOS independently and collectively lead to presentations of metabolic syndrome. In all of the studies, bariatric surgery leads to a significant decrease in BMI for PCOS patients. Much of the peak losses occur approximately 12 months post-surgery. Following weight loss, resolution of metabolic syndrome occurs. In five of the studies, close to all PCOS patients were able to discontinue their medications for dyslipidemia and/or hypertension. One study went so far as to explain how weight loss positively affects the interaction between adiponectin and inflammation. However, the benefits seem to be limited despite substantial weight loss because PCOS patients already demonstrate a high inflammatory status that is resistant to weight loss. It should be noted that one study postulated that these improvements did not correlate with the degree of weight loss. However, this is not to undermine the results reported since metabolic changes were achieved even among those with lesser weight loss amounts. Because most studies only followed up an average of one year, it is unknown if the weight loss is maintained long-term. Insufficient follow-up may skew results in regards to the lasting benefits of treating PCOS with bariatric surgery.

Insulin resistance and bariatric surgery

Weight loss secondary to bariatric surgery, more importantly, helps alleviate PCOS patients of insulin resistance. Collectively among the studies that studied insulin resistance in patients, nearly all patients exhibited either total resolution of T2DM or normalization of insulin levels by the end of the study period. As noted by a few articles, this is an important factor in the chronic menstrual dysfunction seen in PCOS patients. Correction of menstrual cycles and ovulation often coincided with resolution of insulin resistance. One study further analyzed post-surgery results and showed that in comparison to control groups, PCOS patients stabilize earlier on, whereas non-PCOS patients continue, thus yielding a greater improvement of metabolic syndrome. Further studies can be done to determine the difference between PCOS-associated insulin resistance versus obesity-related insulin resistance.

Hyperandrogenism and bariatric surgery

Excess insulin stimulates the secretion of androgens from the ovarian thecal cells via LH receptors [33]. Hyperandrogenism is most commonly measured using testosterone and SHBG levels, with one study incorporating precursor hormones (androstenedione and DHEAS). Testosterone is inversely related to SHBG, so as SHBG levels rise, testosterone levels decrease in turn. The natural timeline appears to be an initial increase of SHBG, followed by a subsequent decrease in testosterone levels. Androgen levels significantly decreased within all studies, but total normalization was only achieved in one study.

Hirsutism is a natural comorbidity associated with hyperandrogenism. However, through analyzing the studies, we found that its amelioration does not directly follow either decrease or normalization of hyperandrogenism. No study showed complete resolution of hirsutism, but rather results ranged from insignificant changes in hirsutism profile to 78% resolved. These findings show that hyperandrogenism is not resolved by bariatric surgery alone, and more research needs to be done to determine the mechanism to better improve its resolution.

Ovulatory dysfunction and bariatric surgery

Extra-gonadal aromatization of testosterone leads to increased levels of estrogen. These levels affect upstream feedback loops that result in altered FSH/LH ratios. With this mechanism in effect, patients experience anovulation and resultant irregular menstrual cycles, which leads to phenotype changes in the ovary in the form of PCOS. Five studies reported complete restoration of menstrual cycle. Three of the studies showed that almost all women had a complete restoration, while two studies had non-significant changes at the end of the study period. One of the two studies with non-significant changes only had a follow-up period of six months, so it is possible not enough time passed for significant changes to present itself. Following restoration, many women seeking to become pregnant were successful via natural conception, while a few utilized assisted reproductive techniques. Despite their success, three of the studies discovered little to no change in ovarian volume even with the restoration of their menstrual cycles. The results show that bariatric surgery generally has a beneficial effect on reestablishing regular menstrual cycles. However, even with postoperative pregnancy success, there is no clear, direct link between ovarian morphology normalization and bariatric surgery.

Limitations

Limited access to data from the PubMed database made it difficult to draw full conclusions and perform a full quality assessment in this review (Table 1). The number of studies available regarding the success of surgical intervention for PCOS is greatly lacking, and the sample size is even more limited. The prevalence of PCOS is high among patients, but the number of PCOS patients undergoing bariatric surgery is not of the same level. Diagnostic criteria are also not uniform across all studies, which affects the study population. Although the studies included were mostly RYGB, there are other types of bariatric surgery that have even less literature that studies their effects on PCOS. Follow-up time is not uniform, which can skew results and lead to significant differences between studies. Loss of follow-up and dropouts in already small sample size affect the statistical analysis of the studies.

**Table 1:** Study characteristics BPD, biliopancreatic diversion; RYGB, Roux-en-Y-gastric bypass; SG, Sleeve gastrectomy. 1: BMI; 2: hyperandrogenism; 3: abnormal menstruation; 4: ovulatory dysfunction; 5: hirsutism; 6: type 2 diabetes mellitus/insulin resistance; 7: hypertension; 8: cholesterol.

Reference	Year	Study Design	Sample size (n)	Follow-up time (mo)	Intervention	Outcome(s)
Escobar-Morreale et al. [23]	2005	Prospective	17	12±5	BPD, RYGB	1, 2, 3, 4, 5, 6
Eid et al. [24]	2005	Retrospective	24	27.5±16	RYGB	1, 3, 4, 5, 6, 7, 8
Jamal et al. [25]	2012	Retrospective	20	46.7±35.3	RYGB	1, 3, 4, 5, 6, 7
Legro et al. [26]	2012	Prospective	29	24	RYGB	1, 2, 3, 4, 5
Eid et al. (abstract) [27]	2014	Prospective	14	12	RYGB	1, 2, 3, 5, 6, 7, 8
Wang et al. (abstract) [28]	2015	Prospective	24	24	SG	1, 2, 3, 4, 5
Turkmen et al. [29]	2016	Prospective	13	6	RYGB	1, 2, 3, 4
Abiad et al. [30]	2018	Prospective	22	12	SG	1, 2, 6, 8
Christ and Falcone (abstract) [31]	2018	Retrospective	44	22.8±3.6	Unknown	1, 2, 3, 4, 6, 8
Singh et al. (abstract) [32]	2020	Prospective	18	12	Unknown	1, 2, 3, 4, 5, 6, 7, 8

Future recommendations

Most importantly, a large, multicenter, randomized controlled trial is needed to fully assess the effects of each surgery type on obese PCOS women. The current guidelines of the National Institute of Health advise bariatric surgery for patients BMI>40 kg/m^2^ or BMI>35 kg/m^2^ with severe comorbidities [33]. However, with PCOS affecting multiple demographics, these guidelines might not be sufficient and should be amended. Studies that compare fertility rates among patients after different types of bariatric surgery would also greatly contribute to the understanding of PCOS treatment.

## Conclusions

PCOS is the most common endocrine disorder in reproductive-age women and is the leading cause of infertility in this population. Bariatric surgery has become the leading option for lasting weight loss in obese individuals and has been utilized in a few cases of obese women with PCOS, but we must consider if bariatric surgery is an effective treatment option for PCOS. Bariatric surgery results in effective weight loss in the short term. Patients are able to reduce the prevalence of metabolic syndrome and improve their insulin resistance. Amelioration of hyperandrogenism and its related manifestations restores regular menstrual cycles and aids in successful pregnancies in patients previously struggling to conceive. Ultimately, upon reviewing the results, we recommend a more widespread implementation of bariatric surgery in treatment options for obese women with PCOS. However, more comprehensive studies with longer follow-up time are needed to study the role of bariatric surgery in obese women with PCOS.
